# Acute, High Dose Metformin Therapy at Reperfusion Decreases Infarct Size in the High-Risk Aging Heart

**DOI:** 10.14336/AD.2023.0205

**Published:** 2023-10-01

**Authors:** Lauryn Bates, Meredith Krause-Hauch, Hao Wang, Mohammad Kasim Fatmi, Zehui Li, Qun Chen, Di Ren, Ji Li, Edward J Lesnefsky

**Affiliations:** ^1^Department of Surgery, Morsani College of Medicine, University of South Florida, Tampa, FL, USA.; ^2^Pauley Heart Center, Division of Cardiology, Department of Internal Medicine, Virginia Commonwealth University, Richmond, VA, USA; ^3^Medical Service, Central Virginia VA Health Care System, Richmond, VA, USA

**Keywords:** myocardial infarction, oxidative injury, reperfusion, electron transport chain, mitochondria, NADH: ubiquinone oxidoreductase

## Abstract

Elderly patients (age > 75) sustain larger infarcts with greater mortality from ST elevation myocardial infarcts (STEMI) despite successful reperfusion treatment. Elderly age remains an independent risk despite correction for clinical and angiographic variables. The elderly represent a high-risk population and may benefit from treatment in addition to reperfusion alone. We hypothesized that modulation of cardiac signaling and metabolism with acute, high dose metformin given at reperfusion would exhibit additional cardioprotection. Using a translational aging murine model (22-24-month C57BL/6J mice) of *in vivo* STEMI (45 min artery occlusion with reperfusion for 24 hours); treatment acutely at reperfusion by high dose metformin decreased infarct size and enhanced contractile recovery, demonstrating cardioprotection in the high-risk aging heart.

Elderly patients have an increased prevalence of coronary artery disease placing them at an increased risk of acute myocardial infarction. Despite successful treatment of ST elevation myocardial infarction (STEMI) by reperfusion of the occluded infarct-related artery, elderly patients sustain larger infarcts with greater 30 day mortality [1]. An elderly age (> 75 years) was an independent risk of mortality and persisted despite correction for clinical and angiographic variables [1]. The age-related increase in infarct size and mortality persists despite improvements in reperfusion modality from thrombolysis to primary emergency percutaneous coronary intervention [2]. These findings suggest that the aged human heart has an increased susceptibility to greater injury during the ischemia of STEMI and its reperfusion treatment.

Cardiac injury is increased during ischemia and reperfusion in aged heart experimental models [3, 4]. Mitochondria are key mediators of myocardial injury during the ischemia and reperfusion (I/R) of STEMI and its treatment. The acute, transient modulation of electron transport at the onset of reperfusion decreases infarct size [5], including in the high-risk older murine heart [6]. Although previous studies used inhibitors of complex I of the ETC to provide proof of concept, these agents are of limited translational relevance [6].

Metformin, a biguanide drug, is prescribed to patients with type 2 diabetes. Metformin inhibits complex I of the ETC leading to activation of AMP-mediated protein kinase (AMPK) and downregulation of mechanistic target of rapamycin (mTOR). The acute administration of high dose metformin at the onset of reperfusion protects the adult heart leading to decreased cardiac injury [5], including in the AMPK-kinase dead mouse, supporting mitochondria and complex I as an important contributing mechanism of protection [5]. The decrease in mitochondrial-driven injury also upregulates Nrf2, a factor critical to decrease the inflammatory response, and activates AMPK and inhibits mTORC1, further favoring reduced tissue injury [7] (Fig. 1A). In the current work, the potential of acute, high dose metformin to protect the high-risk older murine heart was studied (Fig. 1A).

**Figure 1.**
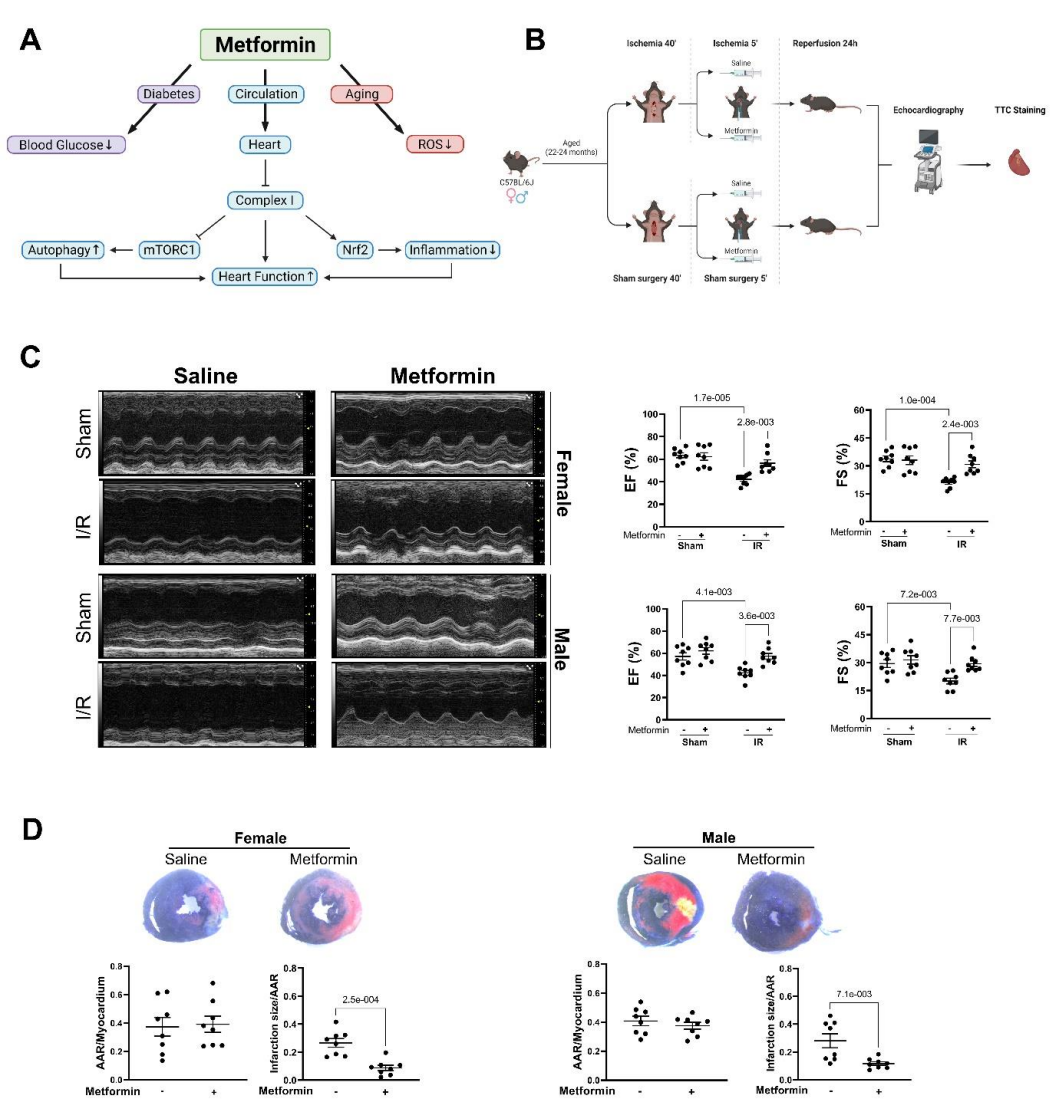
Metformin treatment reduces myocardial infarction. (A) Hypothesis regarding metformin reduction of myocardial reperfusion injury and the effect of metformin on blood glucose levels. (B) Experimental design for study using C57BL/6J mice that received sham operation or I/R injury *via* 45 minutes of left coronary artery reversible ligation with vehicle or 2 mM metformin administered 5 minutes before reperfusion followed by reperfusion for 24 hours. After reperfusion, echocardiography and measurement of infarct size were performed. (C) EF (ejection fraction) and FS (fractional shortening) measured *via* echocardiogram. Left: Representative images of M-mode echocardiography from aged male and female C57BL/6J mice following sham operation or I/R with or without metformin treatment. Right: Quantification of echocardiography measurements. Biological replicates N=8 for each group. (D) Myocardial infarct size was determined using triphenyltetrazolium chloride staining (infarct area-white; viable myocardium at risk-red) and risk area via Evans blue staining (blue). Upper: Representative sections of the extent of myocardial infarction. Lower: Ratio of the area at risk (AAR) to the total myocardial area refers to the myocardium rendered ischemic during coronary ligation, and ratio of the infarcted area (white) to AAR (red) is used to assess the infarct size (N=8 each group).

Aged (22-24 months) C57BL/6J mice, both male and female (provided by the National Institute of Aging), were subjected to *in vivo* I/R surgery via 45 minutes of regional myocardial ischemia. The Animal Care and Use Committees of Virginia Commonwealth University and the University of South Florida approved the study. Five minutes before reperfusion, 2 mM metformin (or an equal volume of saline vehicle) was injected via the jugular vein (Fig. 1B) [5]. Pre- and post-surgery, echocardiography was performed using the Vevo 3100 imaging system. After 24-hours of reperfusion, hearts were excised to determine infarct size and myocardial risk area. Statistical analysis was performed by two-way ANOVA with p<0.05 considered significant. I/R decreased ejection fraction and fractional shortening in aged-C57BL/6J mice for both male and female groups (Fig. 1C). The administration of metformin immediately before reperfusion clearly protected the aged heart in both aged male and female mice. Metformin-treated aged mice demonstrated improved cardiac contractile function (Fig. 1C) and, more importantly, a substantial reduction in infarct size (Fig. 1D). There was no procedure related to mortality in the Sham surgery groups. In the I/R groups, mortality in female mice was similar during reperfusion with 11% mortality in vehicle and metformin groups. In male mice, mortality in I/R groups was 27% during reperfusion in both vehicle and metformin groups. Three mice (two male, one female) died during ischemia before treatment. The sex-based trend in mortality did not meet statistical significance and will require further study.

Acute high dose metformin therapy may assist other proteins, such as Sestrin2, in cardiomyocytes [7], contributing to the activation of AMPK [4, 8] with subsequent downregulation of mTOR [7]. The mitochondria contribute a critical role as mediators of cardiac injury and in regulating cardiac function after I/R [9, 10]. High dose acute metformin treatment modulates the ETC through complex I inhibition effectively achieving cardiac protection in young murine models [5]. Metformin treatment, via enhancement of Sestrin2 [7], contributes to mitochondrial integrity. High dose metformin is effective in decreasing cardiac injury in human cardiomyocytes during hypoxia and reoxygenation [11]. Further studies are needed to assess the potential protection by acute, high dose metformin therapy at the onset of reperfusion in aged translational animal models. The administration of high dose metformin in the setting of human STEMI is potentially feasible [5, 12]. We propose that metformin has potential as an adjunctive catheterization laboratory treatment via intracoronary infusion immediately following reperfusion during direct percutaneous coronary intervention [5, 12] for elderly patients suffering from STEMI to reduce cardiac injury and ideally improve survival.

In summary, the administration of metformin directly before reperfusion significantly improved myocardial salvage following I/R injury in aged mice. Our study suggests that pharmacological metformin treatment could be beneficial for aging STEMI human hearts to increase cardiac function and longevity and provide a therapeutic approach targeted to a specific age-enhanced disease state.

## References

[1] Lesnefsky EJ, Lundergan CF, Hodgson JM, Nair R, Reiner JS, Greenhouse SW, et al. (1996). Increased left ventricular dysfunction in elderly patients despite successful thrombolysis: the GUSTO-I angiographic experience. J Am Coll Cardiol, 28:331-337. 8800106 10.1016/0735-1097(96)00148-9

[2] Newell MC, Henry JT, Henry TD, Duval S, Browning JA, Christiansen EC, et al. (2011). Impact of age on treatment and outcomes in ST-elevation myocardial infarction. Am Heart J, 161:664-672. 21473964 10.1016/j.ahj.2010.12.018

[3] Lesnefsky EJ, Gallo DS, Ye J, Whittingham TS, Lust WD (1994). Aging increases ischemia-reperfusion injury in the isolated, buffer-perfused heart. J Lab Clin Med, 124:843-851. 7798799

[4] Quan N, Sun W, Wang L, Chen X, Bogan JS, Zhou X, et al. (2017). Sestrin2 prevents age-related intolerance to ischemia and reperfusion injury by modulating substrate metabolism. FASEB J, 31:4153-4167. 28592638 10.1096/fj.201700063R PMC5572689

[5] Mohsin AA, Chen Q, Quan N, Rousselle T, Maceyka MW, Samidurai A, et al. (2019). Mitochondrial Complex I Inhibition by Metformin Limits Reperfusion Injury. J Pharmacol Exp Ther, 369:282-290. 30846619 10.1124/jpet.118.254300 PMC6474909

[6] Chen Q, Ross T, Hu Y, Lesnefsky EJ (2012). Blockade of electron transport at the onset of reperfusion decreases cardiac injury in aged hearts by protecting the inner mitochondrial membrane. J Aging Res, 2012:753949. 10.1155/2012/753949 PMC3347723 22619720

[7] Ren D, He Z, Fedorova J, Zhang J, Wood E, Zhang X, et al. (2021). Sestrin2 maintains OXPHOS integrity to modulate cardiac substrate metabolism during ischemia and reperfusion. Redox Biol, 38:101824. 33316744 10.1016/j.redox.2020.101824 PMC7734306

[8] Quan N, Wang L, Chen X, Luckett C, Cates C, Rousselle T, et al. (2018). Sestrin2 prevents age-related intolerance to post myocardial infarction via AMPK/PGC-1alpha pathway. J Mol Cell Cardiol, 115:170-178. 29325933 10.1016/j.yjmcc.2018.01.005 PMC5820139

[9] Lesnefsky EJ, Chen Q, Hoppel CL (2016). Mitochondrial Metabolism in Aging Heart. Circ Res, 118:1593-1611. 27174952 10.1161/CIRCRESAHA.116.307505 PMC5009371

[10] Lesnefsky EJ, Chen Q, Tandler B, Hoppel CL (2017). Mitochondrial Dysfunction and Myocardial Ischemia-Reperfusion: Implications for Novel Therapies. Annu Rev Pharmacol Toxicol, 57:535-565. 27860548 10.1146/annurev-pharmtox-010715-103335 PMC11060135

[11] Emelyanova L, Bai X, Yan Y, Bosnjak ZJ, Kress D, Warner C, et al. (2021). Biphasic effect of metformin on human cardiac energetics. Transl Res, 229:5-23. 33045408 10.1016/j.trsl.2020.10.002 PMC10655614

[12] Chen Q, Lesnefsky EJ (2021). Metformin and myocardial ischemia and reperfusion injury: Moving toward "prime time" human use? Transl Res, 229:1-4. 33148475 10.1016/j.trsl.2020.10.006

